# An Experimental Approach to Rigorously Assess Paneth Cell α-Defensin (*Defa*) mRNA Expression in C57BL/6 Mice

**DOI:** 10.1038/s41598-019-49471-9

**Published:** 2019-09-11

**Authors:** Patricia A. Castillo, Eric B. Nonnecke, Daniel T. Ossorio, Michelle T. N. Tran, Stephanie M. Goley, Bo Lönnerdal, Mark A. Underwood, Charles L. Bevins

**Affiliations:** 1 Department of Microbiology & Immunology, School of Medicine, University of California Davis, Davis, CA 95616 USA; 2 Department of Nutrition, College of Agricultural and Environmental Sciences, University of California Davis, Davis, CA 95616 USA; 3 Department of Pediatrics, School of Medicine, University of California Davis, Sacramento, CA 95817 USA

**Keywords:** Peptides, Innate immunity, Mucosal immunology

## Abstract

Abundant evidence from many laboratories supports the premise that α-defensin peptides secreted from Paneth cells are key mediators of host-microbe interactions in the small intestine that contribute to host defense and homeostasis. α-defensins are among the most highly expressed antimicrobial peptides at this mucosal surface in many mammals, including humans and mice; however, there is striking variation among species in the number and primary structure of α-defensin paralogs. Studies of these biomolecules *in vivo* are further complicated by striking variations between laboratory mouse strains. Herein, we report an experimental approach to determine with precision and specificity expression levels of α-defensin (*Defa*) mRNA in the small intestine of C57BL/6 mice through an optimized set of oligonucleotide primers for qRT-PCR assays and cloned cDNA plasmids corresponding to the *Defa* paralogs. This approach demonstrated marked differences in α-defensin expression in C57BL/6 mice with respect to proximal/distal anatomical location and developmental stage, which have not been described previously. These data underscore the importance of careful attention to method (primer choice, proximal vs. distal location, and developmental stage) in analysis of antimicrobial peptide expression and their impact.

## Introduction

The intestinal mucosa of mammals is a site of remarkably complex and vitally important host-microbe interactions^1^. The intestinal tract harbors a diverse, abundant and dynamic group of microorganisms, which can have profound effects on host nutrition, physiology and immune response^2–9^. While the host derives many benefits from mutualistic interactions with various gut microbes, disruption of this community, termed dysbiosis, creates niche opportunities for the proliferative expansion of noxious organisms that compromise homeostasis and can cause disease^9–23^.

Accumulating evidence indicates that the host orchestrates mucosal homeostasis by accommodating and shaping the composition of contiguous microbial populations^16,24–27^. In the mammalian small intestine, one influential host factor of this ecosystem is a collection of proteins and peptides secreted by Paneth cells^28–30^. In humans, mice, and many other mammals Paneth cells are specialized epithelial cells located at the base of the crypts of Lieberkühn in the small intestine interdigitated between epithelial stem cells^30^. These secretory cells produce especially high quantities of antimicrobial peptides called α-defensins^29–31^. In mice, α-defensins were originally named “cryptdins” (for crypt defensins), which are orthologs of the human α-defensins HD5 (DEFA5) and HD6 (DEFA6)^28,32^. Paneth cell α-defensins are stored in cytoplasmic secretory granules together with other luminally delivered antimicrobials^29^. Secreted α-defensins function to defend the intestinal mucosa from virulent microbes^33,34^, inhibit microbial translocation across the epithelium^35^ and help shape the composition of the colonizing microbiota^24^. Thus, Paneth cell α-defensins serve critically important functions in homeostasis and host defense.

Mice are the most widely studied species to model acute and chronic disease^36^. Many valuable insights on enteric host-microbe interplay have stemmed from, and depend on, investigation of mouse models. Yet, while the *in vivo* dynamics of host-microbe interactions in the intestine can be profoundly influenced by Paneth cell α-defensins, their characterization and biological analysis have been technically challenging due, in part, to their extensive repertoire within and across common laboratory mouse strains. Analysis of the α-defensin gene locus in C57BL/6 mice identified a multitude of *Defa* genes, *Defa*-related genes and *Defa* pseudogenes that share very similar sequences^37,38^. The remarkable number of closely related α-defensin gene paralogs in C57BL/6 mice renders analysis of expression of individual α-defensin mRNA and protein difficult by routine quantitative reverse transcription-polymerase chain reaction (qRT-PCR) or antibody-based experimental approaches. Given the extensive reliance on C57BL/6 mice for studies probing the influence of host genetics on host-microbe interactions, together with the importance of α-defensins in the intestinal ecosystem, there remains a critical need for a robust and rigorous approach to analyze α-defensin expression. To address this problem, Menendez, *et al*.^39^ made a significant initial advance for the field by identifying seven subgroups of expressed *Defa* genes in C57BL/6 mice. Isolation and structural characterization of the mature α-defensin peptides corresponding to these subgroups had been previously reported by others^38,40^. This categorization enabled Menendez *et al*. to develop a set of PCR primer pairs that could amplify each of these *Defa* subgroups by qRT-PCR^39^.

Several years ago, our laboratory developed a robust qRT-PCR approach using external cDNA standards that readily permits quantitative comparisons of mRNA expression for genes encoding antimicrobial peptides in tissues from diverse specimens and experimental influences^41^. In this approach, we determine the absolute mRNA transcript numbers (based on external standard curves) as the primary data, not simply as a normalized ratio to a “housekeeping” gene (i.e., ΔΔCT)^41^. This approach is a bit more labor intensive, but addresses the recommendations by Bustin^42^, who demonstrated that normalization to “housekeeping” gene mRNA levels can compromise comparisons, or at times, lead to erroneous conclusions since expression levels of certain “housekeeping” genes (especially glyceraldehyde-3-phosphate dehydrogenase (*Gapdh*)) may vary by orders of magnitude among tissue sources, experimental conditions and/or tissue pathology^42,43^.

With these issues in mind, the goals of the current study were to (*i*) clone cDNA templates of α-defensin encoding mRNA subgroups in C57BL/6 mice and develop a set of external standard quantitative curves for RT-PCR analysis, (*ii*) use these *Defa* cDNA templates to determine the specificity of each *Defa* PCR primer pair initially reported by Menendez, *et al*.^39^ and modify the primer sequences to substantially enhance specificity, and (*iii*) use the external standard curves to determine the absolute *Defa* mRNA abundance in C57BL/6 mice with respect to several experimental parameters. The latter included expression along the small intestinal tract, during postnatal development, in mice with genetic knockout of the gene encoding myeloid differentiation primary response 88 (MyD88), and in germ-free mice, as well as in mice following streptomycin treatment. We report the development of an optimized set of PCR primers and cloned cDNA standards, which together provide a set of qRT-PCR assays that are quantitative and specific for the paralog subgroups of α-defensin genes in C57BL/6 mice (now expanded to eight). Using these assays, our data extend the previous reports of partial characterization of α-defensin gene expression in this important mouse model. Importantly, our data reveal patterns of α-defensin expression that vary dramatically in terms of anatomical location and developmental stage, which have not been previously elucidated. In addition, our results identify some key potential pitfalls that could arise from interpreting data from less rigorous analytical approaches.

## Results

### Sequence comparisons of *Defa* gene paralogs in C57BL/6 mice

**Figure 1:**
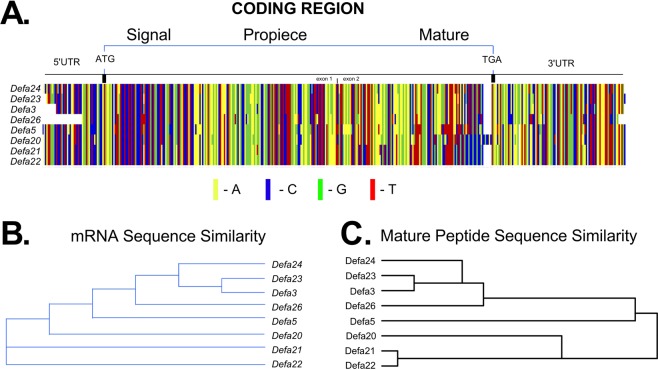
Sequence comparisons of C57BL/6 *Defa* mRNA and mature peptides. (**A**) Sequence alignment of eight *Defa* mRNA paralogs in C57BL/6 mice. The eight *Defa* mRNA paralogs are index members of the subgroups whose sequence alignment is presented in Supplementary Fig. S1. Accordingly the subgroups are: *Defa3* (*Defa3* and *Defa17*), *Defa5* (*Defa5*, *Defa34*, *Defa35*, *Defa36* and *Defa37*), *Defa20* (*Defa20*, *Defa32*, *Defa33*, and *Defa2*), *Defa21* (single member), *Defa22* (single member), *Defa23* (*Defa23*, *Defa27*, and *Defa31*), *Defa24* (*Defa24* and *Defa30*) and *Defa26* (*Defa26* and Gm15292). A high degree of sequence similarity uniformly distributed across coding and untranslated regions are evident. (**B**) Clustal similarity dendrogram of *Defa* mRNA sequences. NCBI accession numbers from sequences used in this analysis are listed in Supplementary Fig. S1. (**C**) Clustal similarity dendrogram of *Defa* mature peptide sequences. Sequences are from the literature^38,40^. Clustal analysis was performed by MacVector software using an unweighted pair group method with arithmetic mean.

The α-defensin gene paralogs in C57BL/6 mice encode a collection of α-defensin mRNA with remarkable sequence similarity (Fig. 1A,B and Supplementary Fig. S1)^38,40^. We refined the paralog grouping proposed by Menendez, *et al*.^39^ to include eight subgroups (Supplementary Table S1): *Defa3* (*Defa3* and *Defa17*), *Defa5* (*Defa5*, *Defa34*, *Defa35*, *Defa36* and *Defa37*), *Defa20* (*Defa20*, *Defa32*, *Defa33*, and *Defa2*), *Defa21* (single member), *Defa22* (single member), *Defa23* (*Defa23*, *Defa27*, and *Defa31*), *Defa24* (*Defa24* and *Defa30*) and *Defa26* (*Defa26* and Gm15292). We were able to establish discriminating assays for *Defa21* and *Defa22*, yielding eight rather than the seven previously described subgroups^39^. The individual members within each group had one or more discriminating nucleotides, but in the case of nonsynonymous differences, the encoded amino acid differences were subtle (Supplementary Fig. S1). Those amino acid substitutions were generally in the signal sequence or propeptide regions, except for *Defa24* vs. *Defa30* (which encode a conservative Leu/Met amino acid difference in the mature peptide), and Defa23 vs. Defa 27 (which encode conservative Ile/Val, Leu/Met, and Met/Ile amino acid differences in the mature peptide). Sequence similarity clustering (Clustal analysis) of the *Defa* mRNA sequences (Fig. 1B) and mature α-defensin peptide sequences (Fig. 1C) show nearly identical patterns for these subgroups.

### Development of new quantitative assays to assess C57BL/6 *Defa* mRNA expression with high specificity

**Figure 2:**
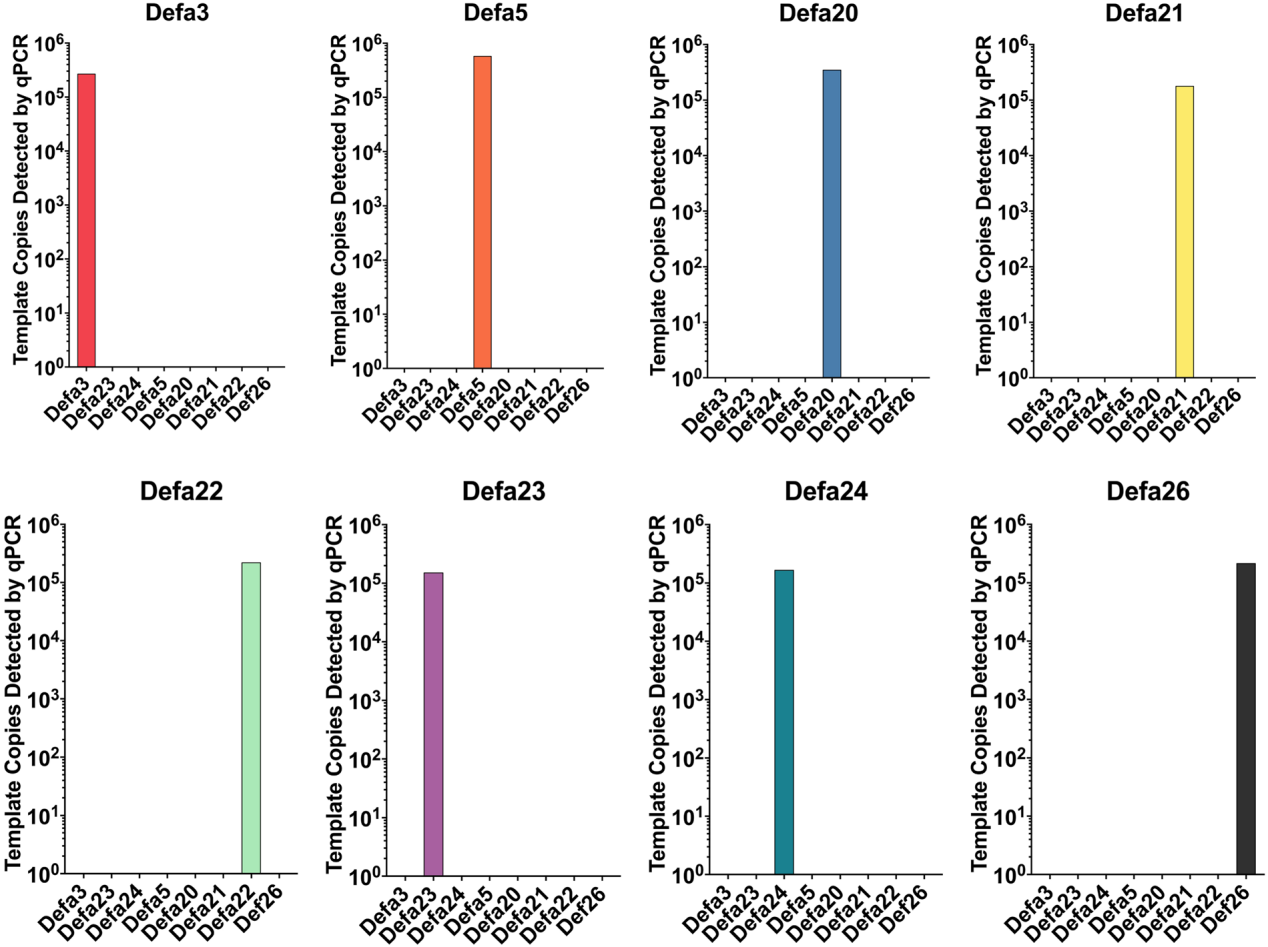
Analysis of specificity for the *Defa* qRT-PCR primer pairs. The eight pairs of *Defa*-targeting oligonucleotide primer pairs (Table 1) were used to amplify each of the eight plasmid cDNA standards present at 10^5^ copies per each reaction tube. Each panel of data represents reactions using the single pair of PCR primers indicated, and the x-axis identifies the cloned cDNA templates used in the eight reactions. The plasmid cDNA standards were cloned, sequenced, and quantitated as described in the methods.

In order to quantitate the expression of each of the eight C57BL/6 *Defa* paralog subgroups, we sought to determine absolute mRNA copy number using methodology previously published by our laboratory^41^. For this approach, we first cloned cDNA for the index member of each of the C57BL/6 *Defa* target paralog subgroups in order to develop standard curves for quantitative measure and rigorous assessment of assay specificity (see Methods). The serially diluted standard cDNAs for each *Defa* subgroup were then used as templates for qRT-PCR amplification using the gene-specific primers (Table 1). These PCR reactions were monitored to determine the threshold of product detection for each concentration of standard template and the data were used to create a standard curve to extrapolate values from experimental tissue samples. In order to ascertain if the *Defa* PCR primers (Table 1) were specific for their designated target, we tested each primer pair against the full panel of cDNA plasmid standards at a template concentration of 10^5^ copies per reaction (Fig. 2). Since each PCR primer pair showed target specificity by amplifying only their intended cDNA target, we conclude that intended- vs. off-target amplification was ≥10^5^-fold in each case.

**Table 1:** Oligonucleotide primer sequences used for qRT-PCR analysis of α-defensin (*Defa*) gene expression in C57BL/6 mice.

Gene Target	Primer Sequence	Product Length (BP)	Annealing Temp (°C)	Reference or Source
*Defa3*	F: ATCTGGTATGCTATTGTAGAAA R: GTGGCCTCAGTACTCATGT	147	62	this work
*Defa5*	F: TCAAAAAAGCTGATATGCTATTG R: AGCTGCAGCAGAATACGAAAG	106	58	F: ref.^39^ R: this work
*Defa20*	F: GAGAGATCTGATATGCTATTG R: AGAACAAAAGTCGTCCTGAG	86	62	F: this work R: ref.^39^
*Defa21*	F: GAGAGATCTGATCTGCCTTTG R: CCTCTATTGCAGCGACGA	45	64	F: ref.^39^ R: this work
*Defa22*	F: AGCAGCCAGGGGAAGAG R: CCTCTATTGCAGCGACGT	124	64	this work
*Defa23*	F: TCTGGTATGCTATTGTAGAAC R: GACAGCAGAGCGTGTATA	95	62	this work
*Defa24*	F: GATCTGGTATGCTATTGTAGAG R: GACAGCAGAGCATGTACAA	97	64	this work
*Defa26*	F: ATTGTAGAAAAAGAGGCTGTAC R: AGCAGAGTGTGTACATTAAATG	81	62	ref. ^39^

PCR reaction conditions were 95 °C for 5 min, followed by 45 cycles of: denaturation at 95 °C for 20 sec, annealing for 20 sec at the temperature specified, and extension at 72 °C for 30 sec. As indicated, the sequences were either from this study or Menendez, *et al*.^39^. F (forward/sense primer), R (reverse/antisense primer).

For analysis of most target genes, selection of PCR primers from adjacent exons allows easy discrimination of amplification from cDNA vs. (contaminating) genomic DNA templates, since genomic DNA will require less efficient amplification across an intron; however, the high sequence similarity of the *Defa* mRNA transcripts (Fig. 1) limited the selection possibilities for discriminatory gene-specific primers. Therefore, the PCR primer pairs for all except *Defa*22 were located within a single exon, giving rise to the possibility that trace contaminating genomic DNA could affect estimates of *Defa* mRNA expression at sites with low *Defa* gene expression (Supplementary Fig. S2). To examine this potential caveat, we treated isolated RNA specimens from colon, a tissue with negligible *Defa* gene expression, with DNase prior to cDNA synthesis. The apparent trace expression in the colon for *Defa*3, *Defa*5 and *Defa*21 (<10e3 mRNA copies per 10 ng mRNA) was eliminated by DNase treatment (Supplementary Fig. S2). In parallel, estimates of beta-actin (*Actb*) expression, whose PCR primer pair spanned an intron (Table 2), did not change after DNase treatment of the colonic RNA supporting that the DNase treatment did not affect the quality of the RNA used in the analysis (Supplementary Fig. S2). Since *Defa* expression in the colon was either decreased or undetectable following DNase treatment, we conclude that the apparent low-level expression without DNase treatment is erroneous, and likely due to trace genomic DNA contamination of the isolated RNA specimens. By contrast, analysis of distal small intestine where *Defa* expression was abundant, showed DNase treatment had no detectable effect on estimates of *Defa* expression values (Supplementary Fig. S2). These control experiments highlight that caution is needed when interpreting and reporting low level mRNA expression (≤10e3 mRNA copies per 10 ng mRNA) using most primers in Table 1. Our data indicate that either intron-spanning PCR primer pairs (*Defa22*) and/or DNase pretreatment of RNA is warranted to draw rigorous conclusions on low-level expression. Several additional control experiments for rigor and reproducibility of this qRT-PCR experimental approach are described in the Materials and Methods, and others were reported by our group previously^41^.

**Table 2:** Oligonucleotide primer sequences used for qRT-PCR analysis of gene expression in C57BL/6 mice.

Gene	Primer Sequence	Product Length (BP)	Annealing Temp	Reference/Source
*Actb*	F: GCTGAGAGGGAAATCGTGCGTG R: CCAGGGAGGAAGAGGATGCGG	99	58	ref.^77^
*Lyz1*	F: GCCAAGGTCTACAATCGTTGTGAGTTG R: CAGTCAGCCAGCTTGACACCACG	86	58	ref.^77^
*Lyz2*	F: GGCTGGCTACTATGGAGTCAGCCTG R: GCATTCACAGCTCTTGGGGTTTTG	177	58	ref.^46^
*Nod2*	F: CGACATCTCCCACAGAGTTGTAATCC R: GGCACCTGAAGTTGACATTTTGC	123	58	ref.^77^
*Mmp7*	F: TTCAAGAGGGTTAGTTGGGGGACTG R: TTGTCAAAGTGAGCATCTCCGCC	152	58	ref.^46^
*Slc10a2*	F: TTGCCTCTTCGTCTACACC R: CCAAAGGAAACAGGAATAACAAG	107	58	this work

The primer target sequences were in exons so as to span an intron in the genomic sequence. PCR reaction conditions were 95 °C for 5 min, followed by 45 cycles of: denaturation at 95 °C for 20 sec, annealing for 20 sec at 58 °C, and extension at 72 °C for 30 sec. F (forward/sense primer), R (reverse/antisense primer).

### Anatomical expression of Defa mRNAs in the adult C57BL/6 mouse small intestine

**Figure 3:**
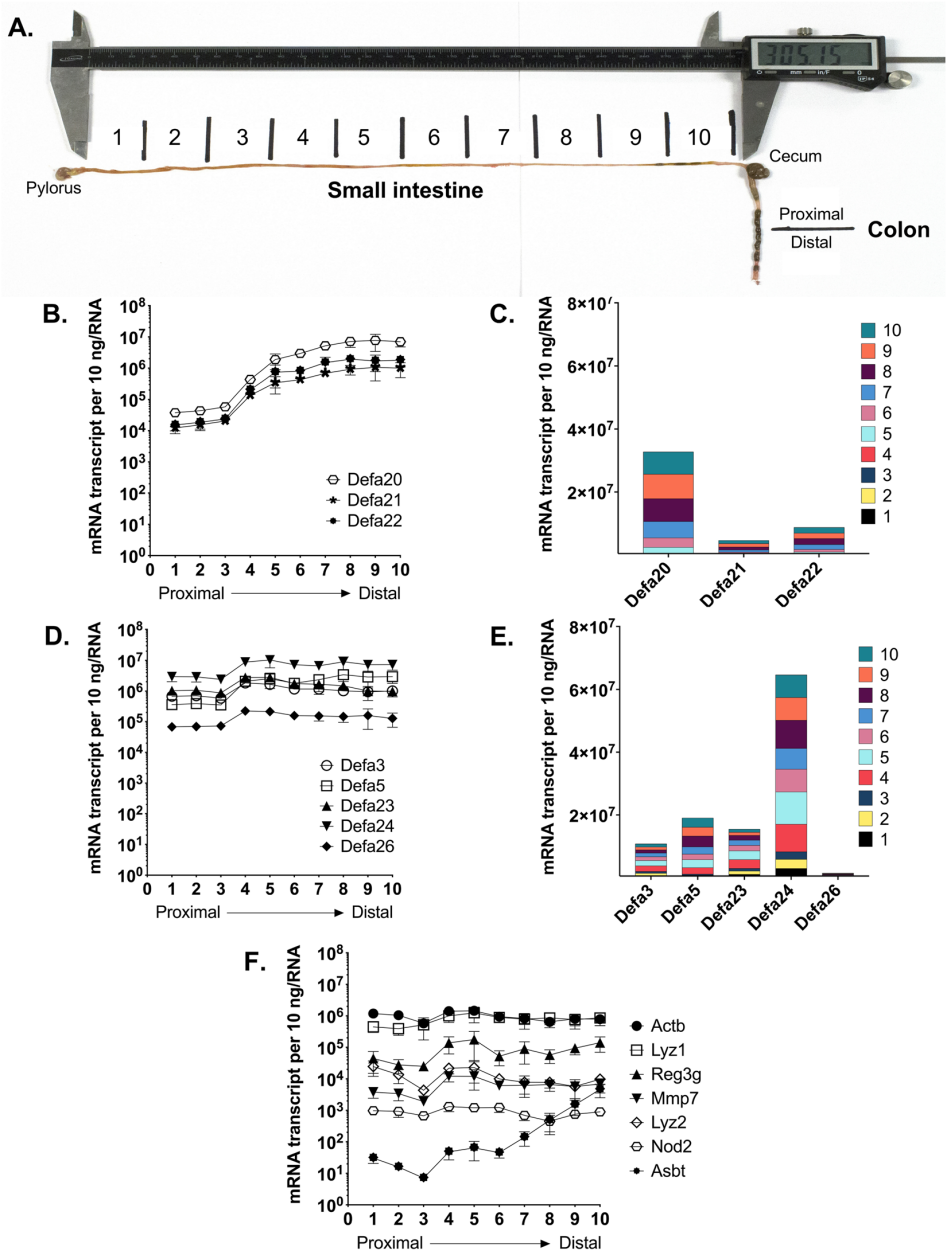
Absolute quantification of *Defa* mRNA in adjacent sections along the small intestinal tract of wild type C57BL/6 mice. (**A**) Image depicts the most proximal to the most distal sections (3 cm each) of small intestine procured for analysis. (**B,D**) Quantitative RT-PCR analysis and (**C,E**) relative percent of each mouse *Defa* mRNA gene analyzed in tissue in 3 cm sections from the most proximal to most distal small intestine. (**F**) Quantitative RT-PCR analysis of other Paneth cell products and control genes in 3 cm sections from the most proximal to the most distal small intestine. PCR oligonucleotide primers were described in Table 1 (for panels B,D) and Table 2 (for panel F). The error bars represent standard error of the mean, N = 4 mice.

Previous studies with FVB and BALB/c mice show regional variations for expression of some Paneth cell α-defensin genes in the small intestine^44–46^. In particular, cryptdin 4^44–46^ and the cryptdin-related sequence 4c (Crs4c/*Defcr-rs10*)^46^ had higher expression in the distal small intestine (ileum) compared to more proximal sites. However, C57BL/6 mice do not express either cryptdin 4 or Crs4c^40,47^ and regional analysis of expression for other α-defensin genes is lacking. Therefore, we sought to determine quantitatively the anatomical expression of each *Defa* gene in adult C57BL/6 mouse small intestine by analyzing adjacent 3 cm sections along the proximal to distal axis, giving a total of ten sections (Fig. 3A). Absolute quantities of each *Defa* mRNA were determined by the qRT-PCR approach outlined above. The data from this analysis (Fig. 3B,D) yielded several interesting findings. *Defa3*, *Defa5*, *Defa23*, *Defa24* and *Defa26* expression were relatively uniform along the tract (Fig. 3D). Among these, *Defa24* showed remarkably high expression (5 × 10^6^–1 × 10^7^ mRNA copies per 10 ng mRNA) in all sections throughout the small intestine (Fig. 3D,E). *Defa*3, *Defa5* and *Defa23* were also abundant, but about 5-fold less than *Defa24* (Fig. 3B,D). In contrast to the uniform expression of those *Defa* genes, *Defa20, Defa21 and Defa22* mRNA expression showed marked regional differences, with dramatically higher expression (approximately 100-fold) in the distal sections (Fig. 3B). *Defa26* was found to be the least abundant among the α-defensin mRNA at all locations (Fig. 3D,E). The patterns of expression for *Defa* mRNA parallel the sequence similarity clustering (Clustal analysis) of mRNA (Fig. 1B) and mature α-defensin peptide sequences (Fig. 1C) highlighting sequence associated patterns of expression.

Expression of other host-microbe related genes in the small intestine were also analyzed in the ten sections, including regenerating islet-derived 3 gamma (*Reg3g*), the two lysozyme paralogs (*Lyz1* (“Paneth cell”) and *Lyz2* (“macrophage”), matrix metalloproteinase-7 (*Mmp7*, the α-defensin processing enzyme in mice^33,34^), and the nucleotide-binding oligomerization domain containing protein 2 (*Nod2*, which recognizes intracellular peptidoglycan), as well as control genes *Actb* and the apical sodium-dependent bile acid transporter (*Slc10a2*). All of these genes, except *Slc10a2* were expressed at a relatively constant level along the small intestine (Fig. 3F). Previous studies by others established that *Slc10a2* is expressed much more prominently in the distal small intestine, compared to proximal sites^48^. The qPCR expression data for *Slc10a2* obtained here (Fig. 3F) reflected that pattern and served as a control to verify the proximal/distal orientation of the intestinal sections.

**Figure 4:**
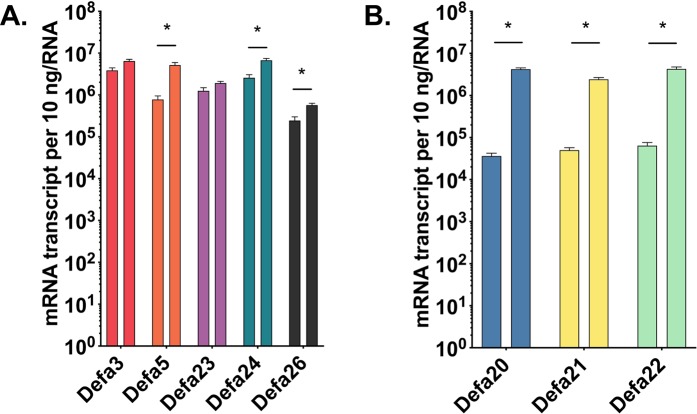
Quantitative comparison of *Defa* mRNA expression in proximal and distal small intestine of wildtype C57BL/6 mice. Quantitative real-time PCR analysis showing absolute quantification of each mouse *Defa* mRNA analyzed in tissue from the most proximal (left bars) and distal 10 cm sections (right bars) of the small intestine of wild type C57BL/6 mice. (**A**) Expression of *Defa3*, *Defa5*, *Defa23*, *Defa24* and *Defa 26* paralog groups which have less pronounced anatomic expression differential. (**B**) Expression of *Defa20*, *Defa21* and *Defa 22* paralog groups which have greater anatomic expression differential. The error bars represent standard error of the mean. A Mann-Whitney test was used for non-parametric statistical analysis (p values less than 0.05 were considered significant, N = 4 mice).

**Figure 5:**
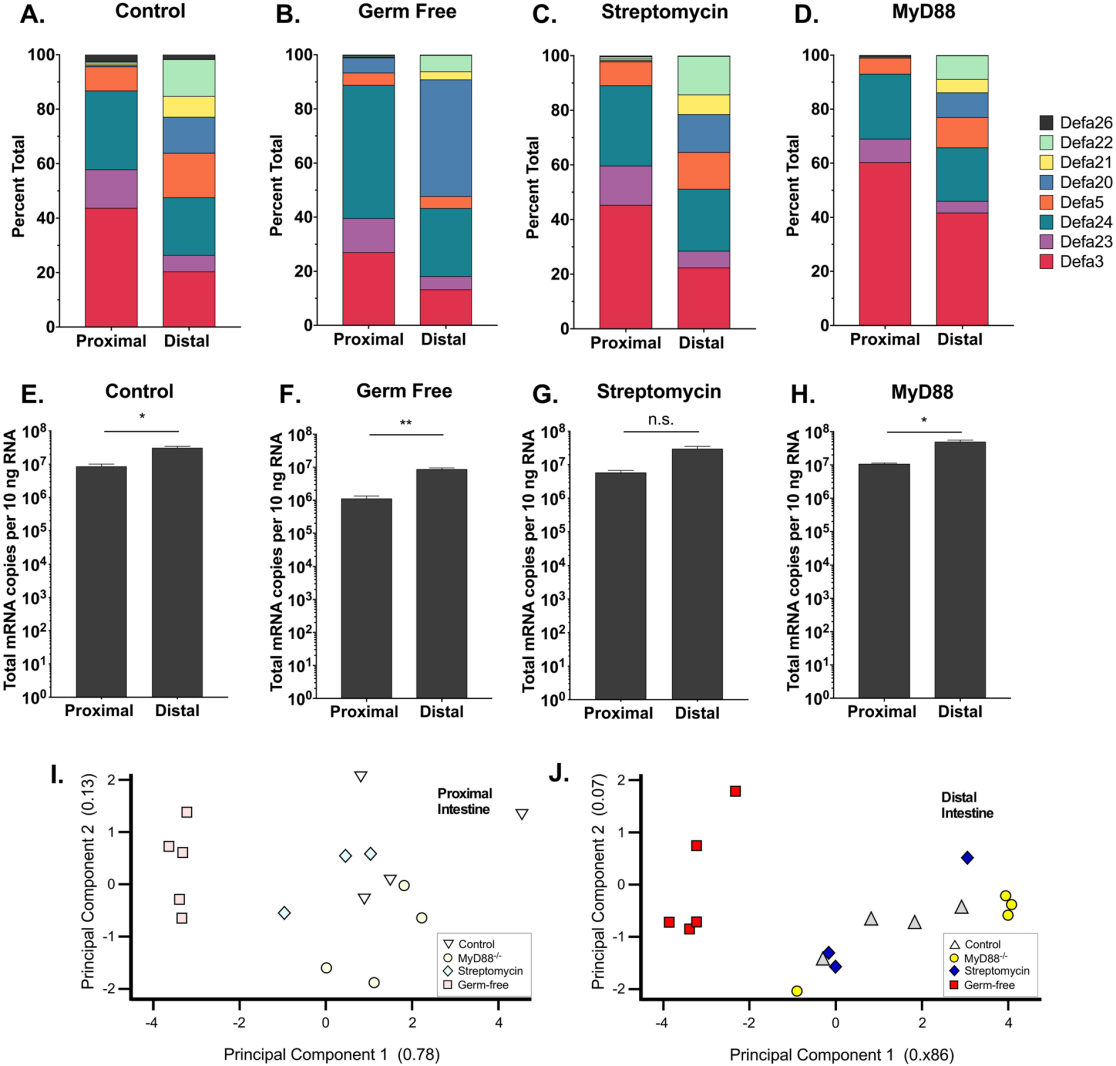
Expression of *Defa* mRNAs in the small intestine C57BL/6 mice. Quantitative real-time PCR analysis showing relative percent of each mouse *Defa* mRNA expressed (**A–D**) and an arithmetic sum of all Defa transcripts detected (**E–H**) in the most proximal and distal 10 cm tissue sections of the small intestine of (**A,E**) wild type control, (**B,F**) germ-free, (**C,G**) streptomycin treated and (**D,H**) *MyD88* gene knockout mice. Weighted principle component analysis of data from A-D for the most proximal (**I**) and distal (**J**) 10 cm sections of the small intestine. For E-H, the error bars represent standard error of the mean. A Mann-Whitney test was used for non-parametric statistical analysis (p values less than 0.05 were considered significant, N = 4 for untreated and MyD88, N = 3 for streptomycin, and N = 5 for germ-free). *p < 0.05, **p < 0.01.

*Defa* expression levels were very similar across the first 3–4 proximal sections; likewise, the most distal 3–4 sections showed similar levels of *Defa* expression (Fig. 3B,D). To simplify subsequent analysis of anatomic expression, we decided to analyze 10 cm of the most proximal and 10 cm of the most distal segments of small intestine. Consistent with data in Fig. 3, *Defa3*, *Defa23*, *Defa24* and *Defa26* were expressed at similar levels in the proximal and distal small intestine (Fig. 4A), while the differentially expressed *Defa* mRNA were more abundant in the distal region (Fig. 4B). Thus, *Defa5* mRNA is 10-fold more abundant, whereas *Defa20*, *Defa21* and *Defa22* are 100-fold more abundant in distal sections. In the proximal small intestine, *Defa* mRNA expression was dominated by *Defa3*, *Defa*5, *Defa23*, *Defa24*, whereas in the distal region the relative *Defa* mRNA expression was more proportionately distributed (Fig. 5A). The sum (total) number of *Defa* transcripts in the distal intestine was ~5-fold higher than in the proximal small intestine (Fig. 5F). No significant differences were detected when comparing data from male and female mice (data not shown).

### *Defa* expression in germ-free and antibiotic treated mice

As compared to conventional mice, mice reared under germ-free conditions have profound alterations in both their innate and adaptive immune systems^49–52^. For example, the expression of *Reg3g* in the small intestinal epithelium of ex-germ-free mice is markedly induced (~10- to 100-fold) upon colonization with a conventional microbiota through a MyD88-dependent mechanism^35,46,53^. Previous studies have indicated that expression of Paneth cell α-defensins has less dependence (~2- to 10-fold) on the presence of a conventional microbiota^40,45,46,53,54^. Using our quantitative approach, the absolute copy number of each *Defa* mRNA was determined in 10 cm sections of the proximal and distal small intestine of adult C57BL/6 mice (Fig. 5). When examining the total abundance of *Defa* mRNA there were significantly fewer *Defa* transcripts (~25-fold) in the small intestine of germ-free mice at both the proximal and distal sites compared to conventionally housed controls (Fig. 5E,F). The relative pattern of abundance of individual *Defa* mRNA in the proximal and distal small intestine appeared similar to that of untreated control mice (Fig. 5A,B). By weighted PCoA, *Defa* expression in germ-free mice clustered away from controls, owing to differences in absolute expression levels (Fig. 5I,J). We conclude that *Defa* mRNA levels are significantly lower in both the proximal and distal small intestine of germ-free mice, but that the relative pattern of *Defa* expression is similar. In control experiments, expression of *Reg3g* in the conventional mice was at levels comparable to *Actb*, whereas in the germ-free animals *Reg3g* expression was lower by approximately 10- to 100 fold (Supplementary Fig. S3), consistent with previous reports^46,53,55,56^.

Since mice are routinely orally gavaged with streptomycin before oral challenges with pathogens such as *S*. Typhimurium to overcome microbiota-mediated resistance to infection^57–60^, we sought to determine if streptomycin treatment modulated *Defa* mRNA expression. Adult C57BL/6 mice were orally gavaged with streptomycin (1 mg/g body weight), which is a typical protocol prior to inoculation with enteropathogens. After four days, the absolute copy number of each *Defa* mRNA was determined in 10 cm sections of the proximal and distal small intestine. No statistically significant differences in *Defa* mRNA expression were detected in streptomycin-treated mice compared to untreated control mice in terms of total abundance of *Defa* mRNA in either the proximal or distal small intestine (Fig. 5G). The relative abundance pattern of the *Defa* mRNA (Fig. 5C) was similar and on weighted PCoA plots, the data points for the specimens from streptomycin-treated mice cluster with untreated controls in both the proximal and distal small intestine (Fig. 5I,J). Thus, while *Defa* mRNA expression varies when comparing germ-free to conventional mice, expression patterns are resilient to less profound transient perturbations in luminal microbes.

### *Defa* expression in *MyD88* knockout mice

MyD88 is an adaptor protein required for signaling from the TLR/IL-1 family of receptors. The expression of *Reg3g* in the small intestine is critically dependent on this biomolecule^35,53,61^. A previous study reported that *MyD88* deficient mice had significantly lower expression of all *Defa* genes (3- to 20-fold) in the distal small intestine^39^, whereas others reported no significant differences in small intestinal *Defa* gene expression in *MyD88* deficient compared to controls^61^. In the present study, absolute copy number of each *Defa* mRNA was determined in both the proximal and distal small intestine of *MyD88* knockout mice (Fig. 5D,H). No statistically significant differences in *Defa* mRNA expression were detected in *MyD88* knockout mice compared to control C57BL/6 mice. Thus, the total abundance of *Defa* mRNA in the proximal and distal small intestine was indistinguishable from control mice (Fig. 5H), the relative abundance pattern for the *Defa* mRNA transcripts (Fig. 5D) was similar, and data points from the MyD88 knockout cluster with data points from control wildtype mice on weighted PCoA plots (Fig. 5I,J). Consistent with previous reports^35,55,61,62^, expression of *Reg3g* in both the proximal and distal small intestine was approximately 10- to 100-fold lower in MyD88 knockout mice compared to wildtype controls (Supplementary Fig. S3).

### Dynamics of *Defa* gene expression during postnatal development of C57BL/6 mice

**Figure 6:**
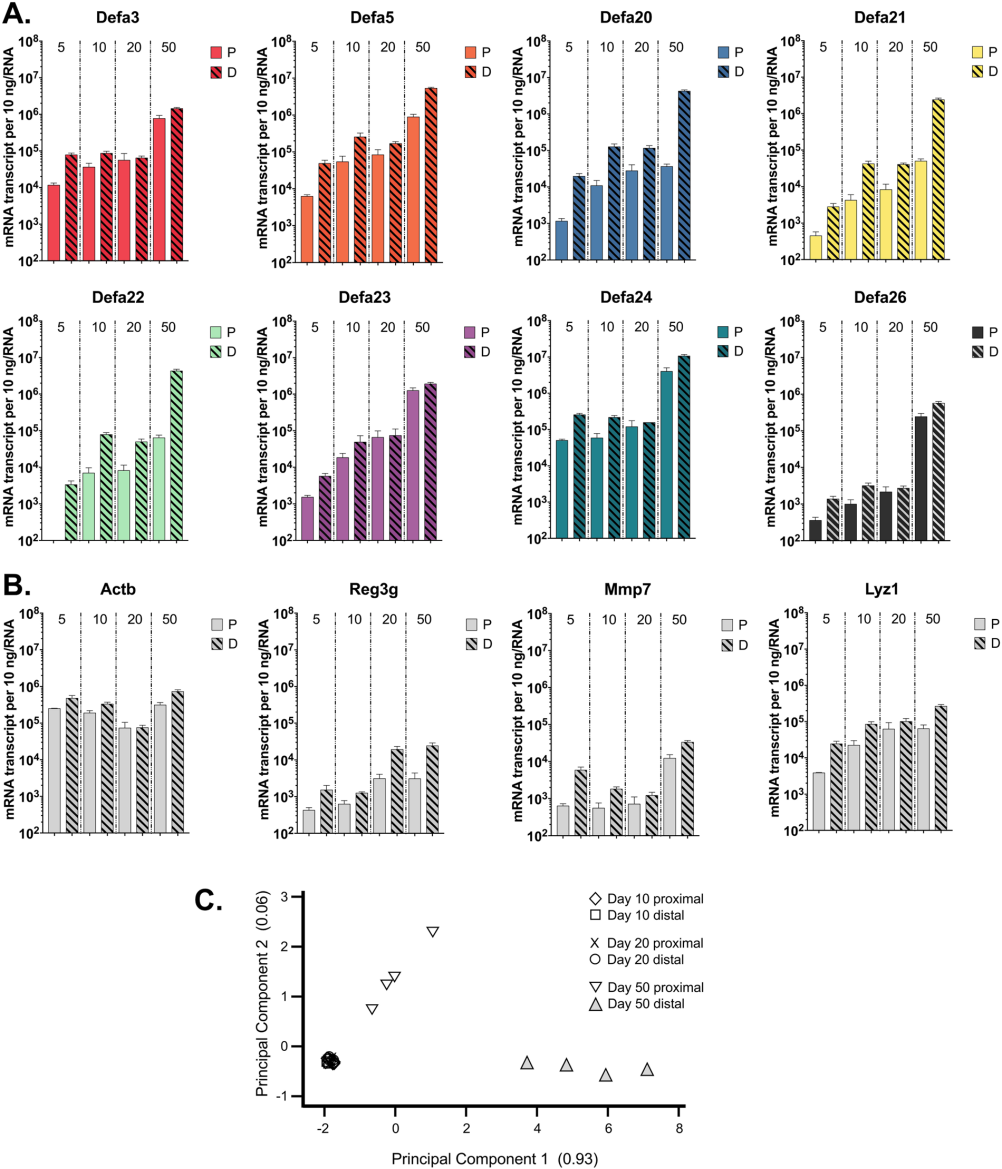
Expression of *Defa* mRNA in the small intestine during development of C57BL/6 mice. (**A**) Quantitative RT-PCR analysis of *Defa* mRNA and (**B**) other Paneth cell products and control genes analyzed in the most proximal 10 cm to the most distal 10 cm sections of the small intestine in post-natal day 5, 10, 20 and 50. Error bars represent standard error of the mean, N = 4 mice. (**C**) Weighted principal component analysis of *Defa* mRNA levels in the most proximal and distal 10 cm of the small intestine on postnatal day 10, 20, 50.

Previous studies using outbred Swiss mice, and inbred FVB and BALB/c mice have shown that *Defa* genes can be detected in neonatal small intestinal tissue and that their expression increases until adulthood^45,63–65^. To address how small intestinal expression of the *Defa* genes change during development using our qRT-PCR approach, we isolated samples of proximal and distal small intestine from pre-weanling (postnatal days 5, 10, and 20) and adult (postnatal day 50) mice. In both proximal and distal small intestine, collective transcript levels of *Defa* genes increased from postnatal days 5 to 50 reaching levels of 10^5^–10^7^ copies per 10 ng RNA in proximal and distal small intestine, respectively (Fig. 6A). The increased abundance was most marked from days 20 to 50 for all *Defa* genes. Increased expression was also observed when comparing days 5 to 10 for all *Defa* genes with the exception of *Defa24* and *Defa3*. More modest changes for all *Defa* genes were observed between postnatal days 10 and 20. Similar to the *Defa* genes, *Reg3g*, and *Lyz1* (Paneth cell lysozyme) exhibited increased expression from postnatal day 5 to 50, with *Reg3g* showing high expression that plateaued by postnatal day 20 (Fig. 6B). However, the magnitude of change was lower for both *Reg3g* and *Lyz1* (~10-fold) compared to the *Defa* genes (~100-fold), in both proximal and distal small intestine. Little change in *Reg3g* and *Lyz1* expression was detected in the colon during development (Supplementary Fig. S4). The expression of *Actb* mRNA was relatively consistent at all anatomic sites and independent of age.

## Discussion

Paneth cell α-defensins mediate critically important functions for the intestinal ecosystem, including helping to shape the composition of the colonizing microbiota, as well as protecting the host from food and water-borne pathogens^28–30^. Determining expression levels of α-defensins can be experimentally challenging, in part, because the significant variation in sequence between mammalian species requires species-specific reagents. Special challenges are encountered with investigations of mice, the most commonly utilized laboratory model for probing host-microbe interaction. Owing to extensive gene duplication in the defensin gene locus, common laboratory mouse strains harbor exceptionally variable collections of α-defensin gene paralogs, often with high sequence similarity^37,38,40,66^. Together these features make the design of discriminating assays difficult. Our approach uses a collection of cloned external standards that permit not only quantitative measure, but also provide a means to directly assess assay specificity. The PCR primer pairs developed here (Table 1) for the qRT-PCR assays were initially based on those reported by Menendez^39^. Our panel of cDNA clones for each of the eight target *Defa* subgroups enabled meticulous optimization of the primer pairs used in this study, which provided target specificity of ≥10^5^-fold compared to off-target PCR amplification (Fig. 2).

One of the important findings reported here is the anatomic distribution of expression along the length of the small intestine (Fig. 3), yielding several notable expression patterns: (*i*) The total abundance of *Defa* mRNA was lower in the most proximal sections; (*ii*) *Defa24* and *Defa20* were the most abundant mRNA in any of sections; (*iii*) Expression of *Defa20*, *Defa21* and *Defa22* mRNA was dramatically (~100-fold) increased in the more distal segments. These regional expression differences are consistent with data from previous studies in outbred Swiss mice^44^, and inbred 129/Sv^65^, BALB/c^45^ and FVB mice^46^, as well as a recent study using single-cell RNA sequence analysis of small intestine in C57BL/6 mice^67^. Higher *Defa* mRNA expression in the distal small intestine could not be attributed solely to numbers of Paneth cells per crypt, which are higher distally by only about 3-fold^63,68^.

An important practical point regarding experimental reproducibility and rigor should be emphasized. Because of the substantial variation in expression of *Defa* genes along the small intestinal tract, careful sample procurement is critically important in experiments aimed to assess *Defa* mRNA abundance between treatment groups. For example, estimates of *Defa20, Defa21 and Defa22* mRNA levels could erroneously *appear* to vary significantly as a result of experimental manipulation, while measured differences could be, in fact, dependent solely on imprecise anatomic sampling since expression levels vary in proximal vs. distal sections ~100- to 1000-fold.

Whereas this investigation focused on small intestinal α-defensin gene expression assessed at the mRNA level, the expression data reported here are supported by previous investigations from two laboratories reporting the isolation and characterization of the small intestinal α-defensin peptides from C57BL/6 mice^38,40^, including studies of *in vitro* antimicrobial activity^38^. Of note, the α-defensins showing the striking pattern of much higher mRNA expression in the distal small intestine, *Defa20*, *Defa21*, and *Defa22* (Figs 3 and 4), encode mature peptides with distinguishable features in primary structure (Fig. 1 and Supplementary Fig. S5). These α-defensins have extended C-termini containing multiple cationic amino acid residues and a reduction in the distance between disulfide-participating Cys(IV) and Cys(V) by three amino acid residues (Supplementary Fig. S5)^38,40^. The overall sequence of mature Defa20, Defa21, and Defa22 peptides are more similar with one another, and differ in sequence from the other five mature α-defensins (Fig. 1 and Supplementary Table S2). These latter five (Defa3, Defa5, Defa23, Defa24 and Defa 26) are more similar with one another than the others and more uniformly expressed. It is likely that the marked differences in expression of the structurally dissimilar α-defensins along the small intestine have implications on their protective and/or homeostatic functions. Genomic sequence analysis using ClustalW nucleotide alignments and Pustell matrix blots could not readily discriminate between these two groups (Supplementary Fig. S6), leaving the mechanism for differential gene expression an open question.

Another notable change in *Defa* gene expression reported here is the pattern during postnatal development, with most notable increases detected for all eight subgroups of *Defa* genes when transitioning from postnatal day 20 (weaning period) to adult mice (Fig. 6). The transcript levels of *Defa* genes increased ~10- to 100-fold during this developmental window, whereas *Reg3g*, *Lyz1* (Paneth cell lysozyme), and *Mmp7* expression levels increased modestly (~5- to 10-fold) during this developmental window. The numbers of Paneth cells per crypt in this developmental window of C57BL/6 mice increase from 3–4 per crypt in the days prior to weaning to 5–6 per crypt in the weeks after weaning^69,70^, so much of the observed increase in mRNA levels is attributable to increased gene expression. These changes in gene expression during development in C57BL/6 mice are in-line with previous studies using outbred Swiss, and inbred FVB and BALB/c mice^45,46,63–65^. The high incidence of bacterial diarrhea as a cause of mortality in newborns and young children is likely due to developmental immaturity of the innate immune system, possibly including α-defensins.

Compared to conventionally reared control mice, the collective total abundance of *Defa* transcripts was lower (~25-fold) in the small intestine of germ-free mice at both the proximal and distal sites (Fig. 5). The numbers of Paneth cells per crypt in germ-free mice is modestly lower than in controls (4.1 vs. 6.4 per crypt, respectively)^69^, so much of the observed difference in mRNA levels is attributable to gene expression. These data are in line with previous studies probing the effects of a germ-free environment on quantitative expression of Paneth cell α-defensins in BALB/c, NMR/KI, 129/Sv and outbred Swiss mice^40,45,46,54^. Whereas chronic exposure to antibiotics affects *Defa* expression^39^, the more subtle and transient perturbations in microbiota associated with the commonly employed oral gavage with streptomycin^71^ in conventionally reared control mice resulted in no perceptible change in α-defensin expression (Fig. 5), although transient subtle alterations at other timepoints cannot be ruled out. In a previous study of C57BL/6 mice with more prolonged antibiotic exposure (streptomycin (450 mg/l) in drinking water for four days), decreased expression of *Defa5* (2-fold), *Defa20* (30-fold) and *Defa23* (4-fold) in the distal small intestine were reported^39^.

We also report data addressing a controversial issue in the literature on *Defa* expression in *MyD88* deficient C57BL/6 mice. Our determination of absolute copy number for each *Defa* mRNA in both the proximal and distal small intestine of *MyD88* knockout mice compared to wildtype controls found no significant differences (Fig. 5). As a control, our analysis of expression of *Reg3g* in both the proximal and distal small intestine showed approximately 10- to 100-fold lower mRNA levels in MyD88 knockout mice compared to controls (Supplementary Fig. S3), consistent with several published reports^35,55,61,62^. Thus, our data on *Defa* expression in *MyD88* deficient C57BL/6 mice are in line with the results of Stockinger, *et al*.^61^, rather than an earlier report of significantly lower *Defa* gene expression in the distal small intestine of *MyD88* deficient mice^39^.

qRT-PCR is a widely utilized method of determining mRNA expression levels and the approach reported here should aid in investigations of host-microbe dynamics in C57BL/6 mice. We concur with the recommendation of Bustin^42^ that housekeeping gene expression levels should be reported relative to total tissue RNA, rather than as a normalizing denominator (i.e., ΔΔCT). When values for target gene mRNA levels are normalized to a housekeeping gene information is lost, comparisons between experiments are compromised and erroneous results can stem from differences in housekeeping mRNA levels that might vary among different tissues or with disease status^42,43^. However, even if the oligonucleotide PCR primers of Table 1 are used in qRT-PCR assays relying on control gene expression (i.e., ΔΔCT) rather than the more elaborate external standard comparators used here, specificity of target *Defa* gene assessment is still possible in C57BL/6 mice. A caveat must be noted, however. Because of the extensive nucleotide similarity within the *Defa* mRNA of C57BL/6 mice (Fig. 1), the discriminating PCR primer pairs that were selected and optimized for this study (Table 1) were contained within a single exon, except for the intron-spanning pair targeting *Defa22*. As highlighted in Supplementary Fig S2, when using the primer pairs that do not span an intron, DNase treatment of RNA prior to cDNA synthesis is warranted in order to draw to draw rigorous conclusions on Defa mRNA levels in extra-small intestinal tissues (e.g., colon) with low-level expression (that is, ~10e3 mRNA copies or less per 10 ng mRNA with external standards, or with relative levels of ~0.001 or less compared to *Actb* expression).

In summary, the methodological approach reported here utilizes external standards and generates assessment of absolute concentrations of specific α-defensin mRNA in experimental tissues. The approach overcomes the technological challenge of highly similar α-defensin paralogs in C57BL6 mice and offers a rigorous means to compare Paneth cell α-defensin mRNA expression in investigations in separate laboratories. In some circumstances, simply using the oligonucleotide primers and conditions presented in Table 1, together with being mindful of the anatomic and developmental expression patterns elucidated here, should provide investigators with higher confidence of rigor in data interpretation.

## Methods

### Mice

The Institutional Animal Care and Use Committee at the University of California, Davis, approved all procedures involving live animals and methods of euthanasia; experiments were performed in accordance with these approved procedures. The genetic background of all mice used in these experiments was C57BL/6. Dr. Andreas Bäumler and members of his laboratory generously provided small intestinal tissue (Figs 4 and 5) from germ-free (Taconic Biosciences, Germantown, NY), streptomycin-treated^72^ and MyD88^−/−^ mice^73^. Tissues for experiments of Figs 3 and 6 were from a specific-pathogen free mouse colony at UC Davis.

### RNA isolation and reverse transcription

The general procedures for RNA isolation and synthesis of cDNA were previously described by our group^41^. Briefly, intestinal tissue samples were dissected immediately after mice were euthanized and placed in RNAlater (~1:10 w/v, Sigma Aldrich, St. Louis, MO). The RNAlater specimen tubes were incubated with gentle rocking overnight at 4 °C, and then stored at −20 °C. For processing, the RNAlater solution was decanted and the tissue was homogenized in guanidine thiocyanate buffer^41,74^. Total RNA was isolated using cesium chloride gradient ultracentrifugation^41^, and then quantified using ultraviolet absorption spectrometry at 260 nm. For cDNA synthesis, 1 to 5 µg of total RNA was reverse transcribed using Superscript II reverse transcriptase (Invitrogen, Carlsbad, CA) using an oligo- (dT)_12–18_ primer^41^. The single-stranded cDNA product was purified using Qiagen PCR purification kit (Qiagen, Valencia, CA), and diluted to 10 ng/µl based on the input concentration of total RNA. Previous control experiments by our group demonstrated that when RNA from a single specimen was used to independently synthesize, isolate and purify the cDNA, the reaction-to-reaction variability was ≤ 15%^41^. Other reproducibility assessments of this approach were previously reported^41^.

In experiments to interrogate the possible role of contaminating DNA as confounding factor in qRT-PCR assays, total RNA (1 µg) was treated with DNase I (1 units, Thermo Fisher Scientific, Waltham, Massachusetts) for 30 minutes at 37 °C and then the enzyme was inactivated according to the manufacturer’s suggestions. The resulting RNA sample was used as a template for reverse transcription as described above.

For analysis of expression during development, intestinal tissues were isolated at postnatal day (PD) 5, 10, 20, and 50. Immediately after euthanasia, the intestine was flushed with PBS to clear luminal contents. For PD5, PD10 and PD20, the small intestine was then sectioned equally into proximal and distal portions and then processed as above. For PD 50 (adult) mice, 10 cm of the most proximal small intestine, 10 cm of the most distal small intestine, the proximal half of the large intestine and the distal half of the large intestine were dissected, and then processed as above.

### Generation of Defa standard curves

Primers that target C57BL/6 *Defa* mRNA (Supplementary Table S3) were designed to amplify the collection of *Defa* cDNAs from small intestinal mRNA in four reactions, each yielding mixed PCR products. The PCR reaction conditions were 95 °C for 5 min, followed by 35 cycles of 95 °C, 30 sec, annealing for 30 sec at 58 °C and extension at 72 °C for 90 sec. Final extension time was 7 min at 72 °C. The resulting PCR product pools were then ligated into a pBluescript II s/k plasmid vector (Agilent Technologies, Stratagene Products Division, La Jolla, CA)^75,76^ and transformed into *E. coli* cells. Multiple clones from each PCR reaction were isolated and the plasmid DNA from the clones was purified and sequenced in both directions to determine the identity of each clone. This yielded eight targeted *Defa* cDNAs for use as external standards (*Defa3*, *Defa5*, *Defa20*, *Defa21*, *Defa22*, *Defa23*, *Defa24*, and *Defa26*). The *Defa* specific plasmids were then quantified by UV spectroscopy, the molar concentration of each *Defa* cDNA insert determined, and then 10-fold serial dilutions were performed using yeast RNA (0.2 µl/µl) as a carrier nucleic acid in the diluent. These dilutions provided templates used in real-time PCR with gene-specific PCR primers (Table 1) in order to generate standard curves for each reaction^41^. Using Avogadro’s constant, the number of *Defa* target sequence copies were calculated for each of the standard curve solutions. The *Defa* gene-specific PCR primer pairs for the eight target cDNAs were designed, based initially on those reported by Menendez *et al*.^39^, and then substantially modified to improve target specificity (Table 1).

### Quantitative real-time RT-PCR of Paneth cell products

Gene-specific oligonucleotide primers (Tables 1 and 2) were synthesized by Invitrogen Life Technologies (Carlsbad, CA). Design of these primers used MacVector software (MacVector, Cary, NC), except for those in Table 1 designated as from Menendez, *et al*.^39^. Real-time PCR was performed using single-stranded cDNA from experimental tissues or gene-specific plasmids (from the standard curve described above) as templates, using the specific oligonucleotide primer pairs and LightCycler FastStart DNA Master SYBR Green 1 reagents (with [MgCl_2_] = 1 mM, final) in a thermocycler equipped with a fluorescence detection monitor (Light Cycler, Roche Diagnostics, Mannheim, Germany) as described^41^. Accumulation of fluorescence signal from intercalation of the SYBR green probe into the PCR products was thereby monitored cycle-by-cycle. Absolute quantification of specific mRNA from tissue was determined by extrapolation of the detection threshold (crossing point) to the crossing point for gene-specific external plasmid standard analyzed within each run^41^. A negative control reaction that omitted template cDNA was included with each set of reactions to check for possible cross-contamination. To confirm PCR amplification of the intended product, melting temperature profile curves of every PCR reaction was determined at the end of each reaction as described^41^.

### Statistical analysis

Error bars represent standard error of the mean. For non-parametric statistical comparisons of data between two groups, a Mann Whitney test was performed using Prism 8 (GraphPad Software, Inc). Stata version 12.1 (Statacorp, College Station TX) was used to generate principal component analyses; scree plots of Eigen values were generated to determine the number of components.

## Supplementary information

Supplementary Figs and Tables
