# Supplementary material for: An Experimental Approach to Rigorously Assess Paneth Cell α-Defensin (Defa) mRNA Expression in C57BL/6 Mice
Source: Sci Rep. 2019 Sep 11;9:13115. doi: 10.1038/s41598-019-49471-9 (PMC6739474; doi:10.1038/s41598-019-49471-9)
Supplement: Supplementary file 1 — Supplementary Figs and Tables [file 41598_2019_49471_MOESM1_ESM.pdf]

Supplementary Figures and Tables

An Experimental Approach to Rigorously Assess Paneth Cell α-Defensin (*Defa*) mRNA Expression in C57BL/6 Mice

Patricia A. Castillo[1], Eric B. Nonnecke[1], Daniel T. Ossorio[1], Michelle Tran[1], Stephanie M. Goley[2], Bo Lönnerdal[2], Mark A. Underwood[3], and Charles L. Bevins[1*]

[1] Department of Microbiology & Immunology, School of Medicine, University of California Davis, Davis, CA 95616

[2] Department of Nutrition, College of Agricultural and Environmental Sciences, University of California Davis, Davis, CA 95616

[3] Department of Pediatrics, School of Medicine, University of California Davis, Sacramento CA 95817

*corresponding author clbevins@ucdavis.edu.

Supplementary Figure 1

A. Defa3 Subgroup

NM007850.2 Defa3
NM001167790.1 Defa17

```
UCCUGCUCACCAAUCCUCCAGGUGACUCCCAGCCAUGAAGACACUAGUCCUCCUCUCUGC
UCCUGCUCACCAAUCCUCCAGGUGACUCCCAGCCAUGAAGACACUAGUCCUCCUCUCUGC
************************************************************

CCUCGUCCUGCUGGCCUUCCAGGUCCAGGCUGAUCCUAUCCAAAACACAGAUGAAGAGAC
CCUCGUCCUGCUGGCCUUCCAGGUCCAGGCUGAUCCUAUCCAAAACACAGAUGAAGAGAC
************************************************************

UAAAACUGAGGAGCAGCCAGGGGAAGACGACCAGGCUGUGUCUGUCUCUUUUGGAGACCC
UAAAACUGAGGAGCAGCCAGGGGAAGACGACCAGGCUGUGUCUGUCUCUUUUGGAGACCC
************************************************************

AGAAGGCUCUUCUCUUCAAGAGGAAUCGUUGAGAGAUCUGGUAUGCUAUUGUAGAAAAAG
AGAAGGCUCUUCUCUUCAAGAGGAAUCGUUGAGAGAUCUGGUAUGCUAUUGUAGAAAAAG
************************************************************

AGGCUGCAAAAGAAGAGAACGCAUGAAUGGGACCUGCAGAAAGGGUCAUUUAAUGUACAC
AGGCUGCAAAAGAAGAGAACGCAUGAAUGGGACCUGCAGAAAGGGUCAUUUAAUGUACAC
************************************************************

ACUCUGCUGUCGCUGAACAUGGAGACCACAGAGGACAAGACGAACAUGAGUACUGAGGCC
ACUCUGCUGUCGCUGAACAUGGAGACCACAGAGGACAAGACGAACAUGAGUACUGAGGCC
************************************************************

ACUGAUGCUGGUGCCUGAUGACUACCUCGCAAUAAAUUGUUCGCAAUAUG
ACUGAUGCUGGUGCCUGAUGACCACCUCGCAAUAAAUUGUUCGCAAUAUGC
********************** ***************************
```

C. Defa20 Subgroup

NM183268.4 Defa20
NM001177521 Defa32
NM001270555 Defa33
NM001195634 Defa2

```
  ACAUUGGGCUCCUGCUCACCAAUUCUCCAGGUGACUCACAGCCAUGAAGACACUUGUC
ACACAUUGGGCUCCUGCUCACCAAUUCUCCAGGUGACUCACAGCCAUGAAGACACUUGUC
ACACAUUGGGCUCCUGCUCACCAAUUCUCCAGGUGACUCACAGCCAUGAAGACACUUGUC
  ACAUUGGGCUCCUGCUCACCAAUUCUCCAGGUGACUCACAGCCAUGAAGACACUUGUC
  **********************************************************

CUCCUCUCUGCCCUCGUCCUGCUGGCCUUCCAGGUCCAGGCUGAUCCUAUCCAAAACACA
CUCCUCUCUGCCCUCGUCCUGCUGGCCUUCCAGGUCCAGGCUGAUCCUAUCCAAAACACA
CUCCUCUCUGCCCUCGUCCUGCUGGCCUUCCAGGUCCAGGCUGAUCCUAUCCAAAACACA
CUCCUCUCUGCCCUCGUCCUGCUGGCCUUCCAGGUCCAGGCUGAUCCUAUCCAAAACACA
************************************************************

GAUGAGGAGACUAAUACUGAGGAGCAGCCAGGGGAGGAGGACCAGGCUGUGUCUGUCUCC
GAUGAGGAGACUAAUACUGAGGAGCAGCCAGGGGAGGAGGACCAGGCUGUGUCUGUCUCC
GAUGAGGAGACUAAUACUGAGGAGCAGGCAGGGGAGGAGGACCAGGCUGUGUCUGUCUCC
GAUGAGGAGACUAAUACUGAGGAGCAGGCAGGGGAGGAGGACCAGGCUGUGUCUGUCUCC
*************************** ********************************

UUUGGAGACCCAGAAGGAUCUGCUCUUCAUGAAAAAUCGUCGAGAGAUCUGAUAUGCUAU
UUUGGAGACCCAGAAGGAUCUGCUCUUCAUGAAAAAUCGUCGAGAGAUCUGAUAUGCUAU
UUUGGAGACCCAGAAGGAUCUGCUCUUCAUGAAAAAUCGUCGAGAGAUCUGAUAUGCUAU
UUUGGAGACCCAGAAGGAUCUGCUCUUCAUGAAAAAUCGUCGAGAGAUCUGAUAUGCUAU
************************************************************

UGUAGAAAAGGAGGCUGCAAUAGAGGAGAACAAGUUUAUGGGACCUGCUCAGGACGACUU
UGUAGAAAAGGAGGCUGCAAUAGAGGAGAACAAGUUUAUGGGACCUGCUCAGGACGACUU
UGUAGAAAAGGAGGCUGCAAUAGAGGAGAACAAGUUUAUGGGACCUGCUCAGGACGACUU
UGUAGAAAAGGAGGCUGCAAUAGAGGAGAACAAGUUUAUGGGACCUGCUCAGGACGACUU
************************************************************

UUGUUCUGCUGCCGCCGCCGCCACCGCCACUGAACAUGCAGAUGACAGAGAUAUGACAAC
UUGUUCUGCUGCCGCCGCCGCCACCGCCACUGAACAUGCAGAUGGCAGAGAUAUGACAAC
UUGUUCUGCUGCCGCCGCCGCCACCGCCACUGAACAUGCAGAUGGCAGAGAUAUGACAAC
UUGUUCUGCUGCCGCCGCCGCCACCGCCACUGAACAUGCAGAUGACAGAGAUAUGACAAC
******************************************** ***************

CAUCAGCUCUGAGGCUACUGAUGCUGGGGCCUGAUAACCACUUCUCAAUAAAUUGUUUGC
CAUCAGCUCUGAGGCUACUGAUGCUGGGGCCUGAUAACCACUUCUCAAUAAAUUGUUUGC
CAUCAGCUCUGAGGCUACUGAUGCUGGGGCCUGAUAACCACUUCUCAAUAAAUUGUUUGC
CAUCAGCUCUGAGGCUACUGAUGCUGGGGCCUGAUAACCACUUCUCAAUAAAUUGUUUGC
************************************************************

AAUAUGC
AAUAUGC
AAUAUGC
AAUAUG
******
```

B. Defa5 Subgroup

NM007851.2 Defa5
NM001177528 Defa34
NM001177481 Defa35
NM001270613 Defa36
NM001177522 Defa37

```
ACACAUUGGGCUCCUGCUCAACAAUUCUCCAGGUGACCCCCAGCCAUGAAGACAUUUGUC
                                             AUGAAGACAAUUGUC
                 UCAACAAUUCUCCAGGUGACCCCCAGCCAUGAAGACAUUUGUC
                 UCACCAAUUCUCCAGGUGACCCCCAGCCAUGAAGACAUUUGUC
                 UCACCAAUUCUCCAGGUGACCCCCAGCCAUGAAGACAUUUGUC
                                             ********* *****

CUCCUCUCUGCCCUUGUCCUGCUGGCCUUCCAGGCCCAGGCUGAUCCUAUCCACAAAACA
CUCCUCUCUGCCCUUGUCCUGCUGGCCUUCCAAGUCCAGGCUGAUCCUAUCCAAAAAACA
CUCCUCUCUGCCCUUGUCCUGCUGGCCUUCCAGGUCCAGGCUGAUCCUAUCCACAAAACA
CUCCUCUCUGCCCUUGUCCUGCUGGCCUACCAGGUCCAGGCUGAUCCUAUCCACAAAACA
CUCCUCUCUGCCCUUGUCCUGCUGGCCUACCAGGUCCAGGCUGAUCCUAUCCACAAAACA
**************************** *** * ****************** ******

GAUGAAGAGACUAAUACUGAGGAGCAGCCAGGGGAAGAGGACCAGGCUGUGUCUAUCUCC
GAUGAAGAGACUAAUACUGAGGAGCAGCCAGGGGAAGAGGACCAGGCUGUGUCUAUCUCC
GAUGAAGAGACUAAUACUGAGGAGCAGCCAGGGGAAGAGGACCAGGCUGUGUCUAUCUCU
GAUGAAGAGACUAAUACUGAGGAGCAGCCAGGGGAAGAGGACCAGGCUGUGUCUAUCUCU
GAUGAAGAGACUAAUACUGAGGAGCAGCCAGGGGAAGAGGACCAGGCUGUGUCUAUCUCU
***********************************************************

UUUGGAGGCCAAGAAGGGUCUGCUCUUCAUGAAGAAUUGUCAAAAAAGCUGAUAUGCUAU
UUUGGAGGCCAAGAAGGGUCUGCUCUUCAUGAAGAAUUGUCAAAAAAGCUGAUAUGCUAU
UUUGGAGGCCAAGAAGGGUCUGCUCUUCAUGAUGAAUUGUCAAAAAAGCUGAUAUGCUAU
UUUGGAGGCCAAGAAGGGUCUGCUCUUCAUGAAGAAUUGUCUAAAAAGCUGAUAUGCUAU
UUUGGAGGCCAAGAAGGGUCUGCUCUUCAUGAAGAAUUGUCUAAAAAGCUGAUAUGCUAU
******************************** ******** ******************

UGUAGAAUAAGAGGCUGCAAAAGAAGAGAACGCGUUUUUGGGACCUGCAGAAAUCUUUUU
UGUAGAAUAAGAGGCUGCAAAAGAAGAGAACGCGUUUUUGGGACCUGCAGAAAUCUUUUU
UGUAGAAUAAGAGGCUGCAAAAGAAGAGAACGCGUUUUUGGGACCUGCAGAAAUCUUUUU
UGUAGAAUAAGAGGCUGCAAAAGAAGAGAACGCGUUUUUGGGACCUGCAGAAAUCUUUUU
UGUAGAAUAAGAGGCUGCAAAAGAAGAGAACGCGUUUUUGGGACCUGCAGAAAUCUUUUU
************************************************************

UUAACUUUCGUAUUCUGCUGCAGCUGAAUAUGCAGAUGACAAAGAUAUGACAACCAUCGG
UUAACUUUCGUAUUCUGCUGCAGCUGAAUAUGCAGAUGACAAAGAUAUGACAACCAUCGG
UUAACUUUCGUAUUCUGCUGCAGCUGAAUAUGCAGAUGACAAAGAUAUGACAACCAUCAG
UUAACUUUCGUAUUCUGCUGCAGCUGAAUAUGCAGAUGACAAAGAUAUGACAACCAUCGG
UUAACUUUCGUAUUCUGCUGCAGCUGAAUAUGCAGAUGACAAAGAUAUGACAACCAUCGG
********************************************************** *

CUCUGAGGCCACUGAUGCUGGGGCCUGAUGAUCACUUCUCAAUAAAUUGUUUGCAAUAUG
CUCUGAGGCCACUGAUGCUGGGGCCUGAUGAUCACUUCUCAAUAAAUUGUUUGCAAUAUG
CUCUGAGGCCACUGAUGCUGGGGUCUGAUGAUCACUUCUCAAUAAAUUGUUUGCAAUAUG
CUCUGAGGCCACUGAUGCUGGGGUCUGAUGAUCACUUCUCAAUAAAUUGUUUGCA
CUCUGAGGCCACUGAUGCUGGGGUCUGAUGAUCACUUCUCAAUAAAUUGUUUGCAAUAUG
*********************** *******************************
```

D. Defa21 Subgroup

NM183253.3 Defa21

```
ACAUUGGGCUCCUGCUCACCAAUCCUCCAGGUGACUCCCAGCCAUGAAGACACUUGUCCU
************************************************************

ACAUUGGGCUCCUGCUCACCAAUCCUCCAGGUGACUCCCAGCCAUGAAGACACUUGUCCU
************************************************************

CCUCUCUGCCCUCAUCCUGCUGGCCUACCAGGUCCAGACUGAUCCUAUCCAAAACACAGA
************************************************************

UGAAGAGACUAAUACUGAGGAGCAGCCAGGGGAAGAUGACCAGGCUGUGUCUGUCUCCUU
************************************************************

UGGAGGCCAAGAAGGAUCUGCUCUUCAUGAAAAAUUGUCGAGAGAUCUGAUCUGCCUUUG
************************************************************

UAGAAAUCGUCGCUGCAAUAGAGGAGAACUAUUUUAUGGGACCUGCGCAGGACCUUUUUU
************************************************************

GCGCUGCUGCCGCCGCCGCCGCUGAGAGUGCAGAUGACAAAGAUAUGACAACCAUCAGCU
************************************************************

CUGAGGCCACUGAUGCUGGGGCCUGAUGAACACUUCUCAAUAAAUUGUUUGCAAUAUGC
***********************************************************
```

E. Defa22 Subgroup

NM207658.4 Defa22

```
ACAUUGGGCUCCUGCUCACCAAUCCUCCAGGUGACUCCCAGCCAUGAAGACACUUGUCCU
************************************************************

CCUCUCUGCCCUCAUCCUGCUGGCCUACCAGGUCCAGACUGAUCCUAUCCAAAACACAGA
************************************************************

UGAAGAGACUAAUACUGAGGAGCAGCCAGGGGAAGAGGACCAGGCUGUGUCUGUCUCCUU
************************************************************

UGGAGGCCAAGAAGGAUCUGCUCUUCAUGAAAAAUUGUCGAGAGAUCUGAUCUGCCUUUG
************************************************************

UAGAAAACGUCGCUGCAAUAGAGGAGAACUAUUUUAUGGGACCUGCGCAGGACCUUUUUU
************************************************************

GCGCUGCUGCCGCCGCCGCCGCUGAGAGUGCAGAUGACAAAGAUAUGACAACCAUCAGCU
************************************************************

CUGAGGCCACUGAUGCUGGGGCCUGAUGAACACUUCUCAAUAAAUUGUUUGCAAUAUGC
***********************************************************
```

F. Defa23 Subgroup

NM001012307.2 Defa23
NM007848 Defa 31
NM001170955.1 Defa27

```
AUUGGGCUCCUGCUCACCAAUCCUCCAGGUGACUCCCAGCCAUGAAGACACUAGUCCUCC
AUUGGGCUCCUGCUCACCAAUCCUCCAGGUGACUCCCAGCCAUGAAGACACUAGUCCUCC
                                         AUGAAGACACUAGUCCUCC
                                         *******************

UCUCUGCCCUCAUCCUGCUGGCCUUCCAGGUCCAGGCUGAUCCUAUCCAAAACACAGAUG
UCUCUGCCCUCAUCCUGCUGGCCUUCCAGGUCCAGGCUGAUCCUAUCCAAAACACAGAUG
UCUCUGCCCUUGCCCUGCUGGCCUCCCAAGUCCAGGCUGAUCCUAUCCAAAACAGAGAUG
**********   *********** *** ************************* *****

AAGAGACUAAAACUGAGGAGCAGCCAGGGAAAGAGGACCAGGCUGUGUCUGUCUCUUUUG
AAGAGACUAAAACUGAGGAGCAGCCAGGGAAAGAGGACCAGGCUGUGUCUGUCUCUUUUG
AAGAGAGUAAAAUUGAUGAGCAGCCAGGGAAAGAAGACCAAGCUGUUUCUGUCUCCUUUG
****** ***** *** ***************** ***** ***** ******** ****

GAGACCCAGAAGGCUCUUCUCUUCAAGAGGAAUCGUUGAGAGAUCUGGUAUGCUAUUGUA
GAGACCCAGAAGGCUCUUCUCUUCAAGAGGAAUCGUUGAGAGAUCUGGUAUGCUAUUGUA
GAGACCCAGAAGGCUCUUCUCUUCAAGAGGAAUCGUUGAGAGAUCUGAUAUGCUAUUGUA
*********************************************** ************

GAACAAGAGGCUGCAAAAGAAGAGAACGCAUGAAUGGGACCUGCAGAAAGGGUCAUUUAA
GAACAAGAGGCUGCAAAAGAAGAGAACGCAUGAAUGGGACCUGCAGAAAGGGUCAUUUAA
GAACAAGAGGCUGCAAAAGAAGAGAACGCCUGAAUGGGACCUGCAGAAAGGGUCAUUUAA
***************************** ******************************

UAUACACGCUCUGCUGUCGCUGAACAUGGAGACCACAGAGGACAAGACGAGCAUGAGUAC
UAUACACGCUCUGCUGUCGCUGAACAUGGAGACCACAGAGGACAAGACGAGCAUGAGUAC
UGUACACGCUCUGCUGCCGCUGA
* ************** ******

UGAGGCCACUGAUGCUGGUGCCUGAUGACCACUUCUCAAUAAAUUGUUCGCAAUAUGC
UGAGGCCACUGAUGCUGGUGCCUGAUGACCACUUCUCAAUAAAUUGUUCGCAAUAUGCU
```

G. Defa24 Subgroup

NM001024225.2 Defa24
NM001177485 Defa31

```
ACACUGAGCUGCUACUCACCAAUCCUCCAGGUGACUCCCAGCCAUGAAGACACUAAUCCU
                              GUGUCUCCCAGCCAUGAAGACACUAAUCCU
                              *** **************************

CCUCUCUGCCCUCGUCCUGCUGGCCUUCCAGGUCCAGGCUGAUCCUAUCCAAAAUACAGA
CCUCUCUGCCCUCGUCCUGCUGGCCUUCCAGGUCCAGGCUGAUCCUAUCCAAAACACAGA
****************************************************** *****

UGAAGAGACUAAAACUGAGGAGCAGCCAGGGGAAGAGGACCAGGCUGUGUCUGUCUCUUU
UGAAGAGACUAAAACUGAGGAGCAGCCAGGGGAAGAGGACCAGGCUGUGUCUGUCUCUUU
************************************************************

UGGAGACCCAGAAGGCGCUUCUCUUCAAGAGGAAUCAUUGAGAGAUCUGGUAUGCUAUUG
UGGAGACCCAGAAGGCUCUUCUCUUCAAGAGGAAUCGUUGAGAGAUCUGGUAUGCUAUUG
**************** ******************* ***********************

UAGAGCAAGAGGCUGCAAAGGAAGAGAACGCAUGAAUGGGACCUGCAGUAAGGGUCAUUU
UAGAGCAAGAGGCUGCAAAGGAAGAGAACGCAUGAAUGGGACCUGCAGUAAGGGUCAUUU
************************************************************

AUUGUACAUGCUCUGCUGUCGCUGAACAUGGAGACCACAGAGGACAAGAUGACCAUGAGU
AAUGUACAUGCUCUGCUGUCGCUGAACAUGGAGACCACAGAGAACAAGAUGACCAUGAGU
* *************************************** ******************

ACUGAGGCCACUGAUGCUGGUGCCUGAUGACCACUUCUCAAUAAAUUGCUUGCAAUAUG
ACUGAGGCCACUGACGCUGGUGCCUGAUGACCACUUCUCAAUAAAUUGCUUGCAAUAUGC
*************** *******************************************
```

H. Defa26 Subgroup

NM001079933.2 Defa26
NM001177487 Gm15292

```
AGGUGACUCCCAGCCAUGAAGACACUUGUCCUCCUCUCUGCCCUUUUCCUGCUGGCCUUC
               AUGAAGACACUUGUCCUCCUCUCUGCCCUUUUCCUGCUGGCCUUC
               *********************************************

CAGGUCCAGGCUGAUCCUAUCCAAAACACAGAUGAAGAGACUAAUACUGAGGUGCAGCCA
CAAGUCCAGGCUGAUCCUAUCCAAAAAACAGAUGAAGAGACUAAUACUGAGGUGCAGCCA
** *********************** *********************************

CAGGAAGAGGACCAGGCUGUGUCUGUCUCCUUUGGAAAUCCAGAAGGCUCUGAUCUUCAA
GAGGAAGAGGAGCAGGCUAUGUCUGUCUCCUUUGGAAAUCCAGAAGGCUCUGAUCUUCAA
 ********** ****** *****************************************

GAAGAAUCGUUGAGAGAUCUGGGAUGCUAUUGUAGAAAAAGAGGCUGUACAAGAAGAGAA
GAAGAAUCGUUGAGAGAUCUGGGAUGCUAUUGUAGAAAAAGAGGCUGCACAAGAAGAGAA
*********************************************** ************

CGCAUUAAUGGGACCUGCAGAAAGGGUCAUUUAAUGUACACACUCUGCUGCCUCUGAACA
CGCAUUAAUGGGACCUGCAGAAAGGGUCAUUUAAUGUACACACUCUGCUGCCUCUGA
*********************************************************

UGGAGACCCCAGAGGACAAGACUUCCAUGAACACUGAGGCCACUGAUGCUGGGGCCUGAU

GUCCACUUCUCAAUAAAUUGUCCGCAG
```

Supplementary Figure S1. Sequence comparisons within eight subgroups of -defensin mRNA in C57BL/6 mice. Clustal sequence alignments for *Defa* mRNA designated by groups *Defa3* (A), *Defa5* (B), *Defa20* (C), *Defa23* (F), *Defa24* (G) and *Defa26* (H), or as sole gene members *Defa21* (D) and *Defa22* (E). These groups were first identified by Menendez et al. (39). NCBI accession numbers are presented with the gene names designated in the NCBI entry. Discriminating nucleotide positions in the mRNA within each group is highlighted with a yellow background. **(A)** Group *Defa3* has two members representing a gene duplication event (39) yielding mRNA with a single differing nucleotide and identical protein products. **(B)** Group *Defa5* has five members with nine discriminating mRNA nucleotides yielding protein products that differ at three residues in the signal sequence, two residues in the propeptide region, but no differences in the mature peptide. **(C)** Group *Defa20* has four members representing gene duplication (39) yielding mRNA with discriminating nucleotides at two internal positions and protein products that differ at one residue in the propeptide region, but no differences in the mature peptide. **(D,E)** Groups *Defa21* and *Defa22* each have a single member. **(F)** Group *Defa23* has three members with *Defa23* and *Defa31* representing gene duplication (39) yielding identical mRNA and protein products, and *Defa27* mRNA contains discriminating nucleotides at seventeen internal positions and protein products that differ at six residues in the propeptide region, and three residues (Ile/Val, Leu/Met, Met/Ile) in the mature peptide. **(G)** Group *Defa24* has two members representing gene duplication (39) yielding mRNA with discriminating nucleotides at seven internal positions and protein products that differ at one residue in the propeptide region and one residue (Leu/Met) in the mature peptide. **(H)** Group *Defa26* has two gene members (39) with mRNA containing discriminating nucleotides at six internal positions and protein products that differ at three residues in the propeptide region, but no differences in the mature peptide.

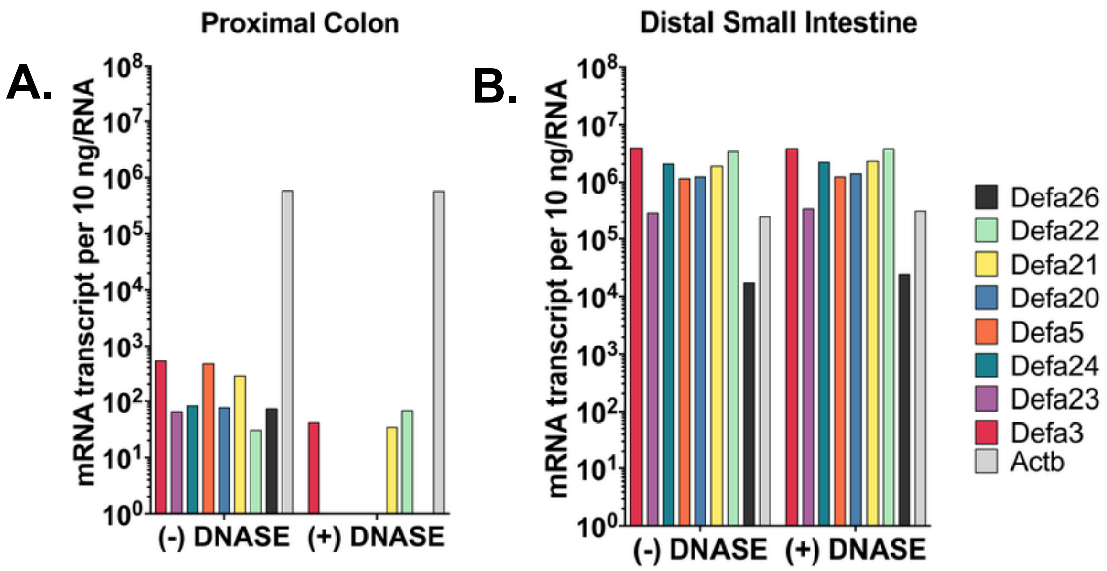

Supplementary Figure 2. DNase treatment of RNA prior to cDNA synthesis eliminates detection of low level expression in the colon and does not affect measures of *Defa* gene expression in the small intestine. (A) Quantitative RT-PCR analysis of mouse *Defa* mRNA in the proximal colon with and without DNase treatment of RNA prior to cDNA synthesis (see methods). Beta-actin (*Actb*) served as a control for RNA integrity with and without DNase treatment, selected because of higher level expression and intron-spanning primers which are features that should minimize any contribution of contaminating genomic DNA in the assay. **(B)** Quantitative real-time PCR analysis of mouse *Defa* mRNA in the distal 10cm of the small intestine with and without DNase treatment (n=1-3 mice).

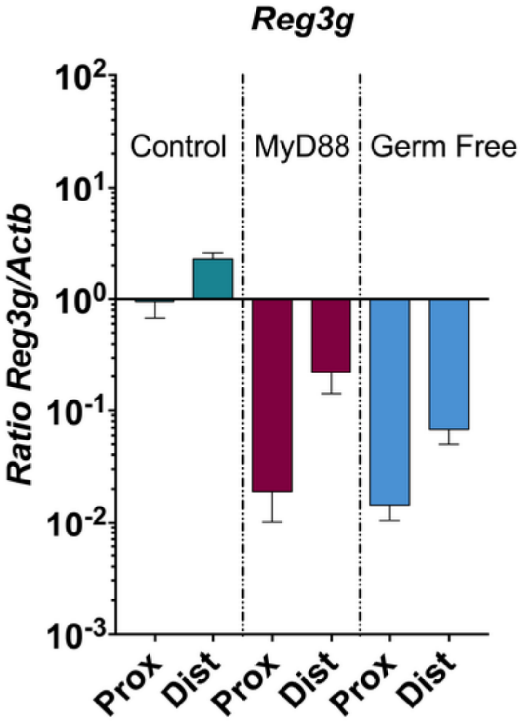

Supplementary Figure 3. Expression of *Reg3g* mRNA in the small intestine of C57BL/6 mice. The expression of *Reg3g* was analyzed by qRT/PCR in the most proximal and distal 10 cm sections of the small intestine of wildtype conventionally housed (control), MyD88 gene knockout and germ-free C57BL/6 mice. The data are presented as a ratio of the absolute values determined for *Reg3g* and *Actb* to facilitate comparisons (n=4-5 mice).

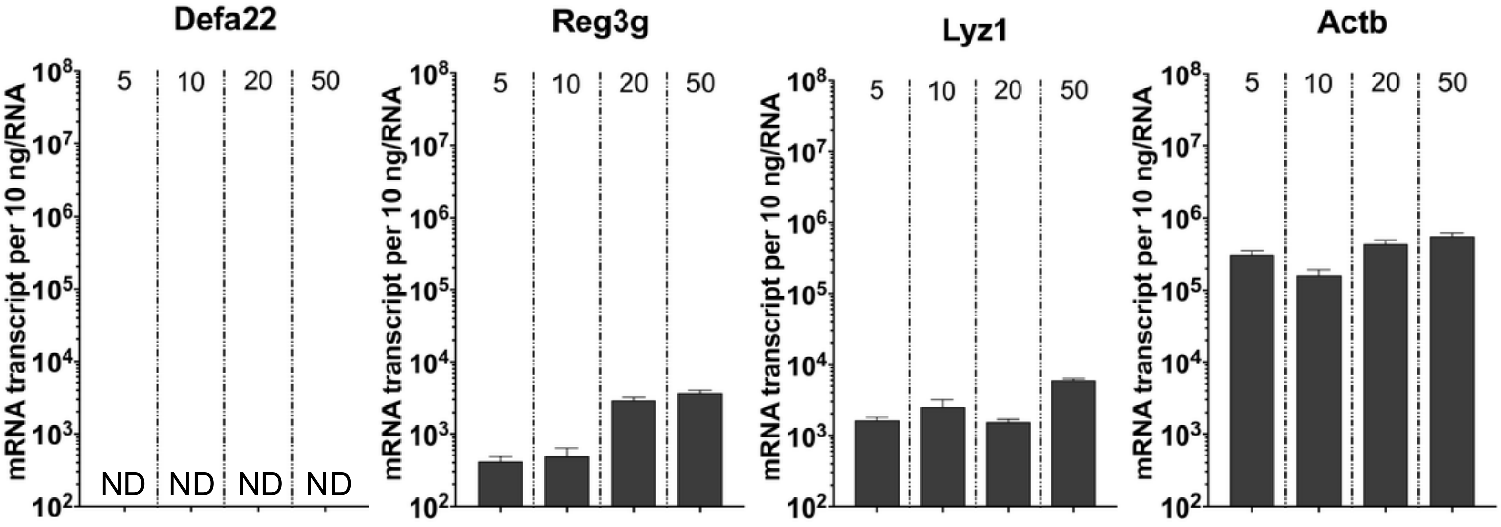

Supplementary Figure 4. Expression of selected mRNA in the colon during development of C57BL/6 mice. Quantitative RT-PCR analysis of *Defa22*, *Reg3g*, *Lyz1*, and *Actb* mRNA in the colon at post-natal day 5, 10, 20 and 50. Error bars represent standard error of the mean, N=4 mice. ND, not detected.

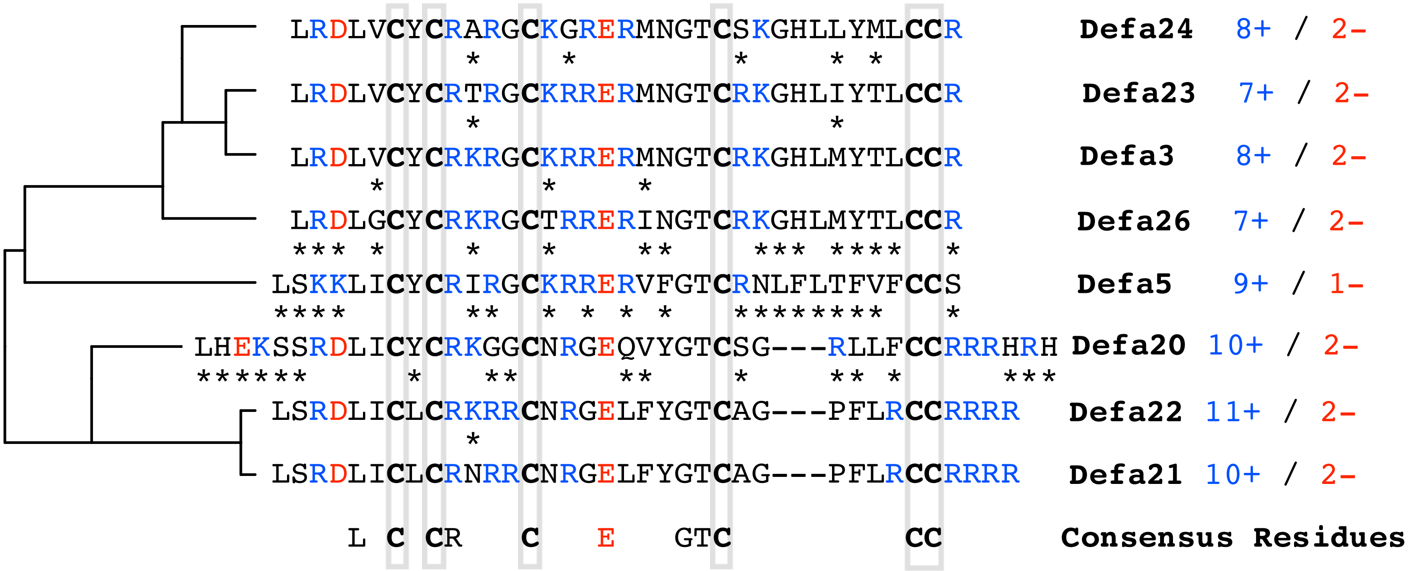

Supplementary Figure 5. Primary structures of *Defa* mature peptide sequences. Sequences are from Shanahan et al [38] and Gulati et al [40]. Discriminating residues between pairs are marked by asterisks. Acid residues are in red and cationic residues in blue. Cysteines that participate in the intramolecular tri-disulfide array are boxed in gray. Clustal analysis associations shown on left was performed as described in Figure 1.

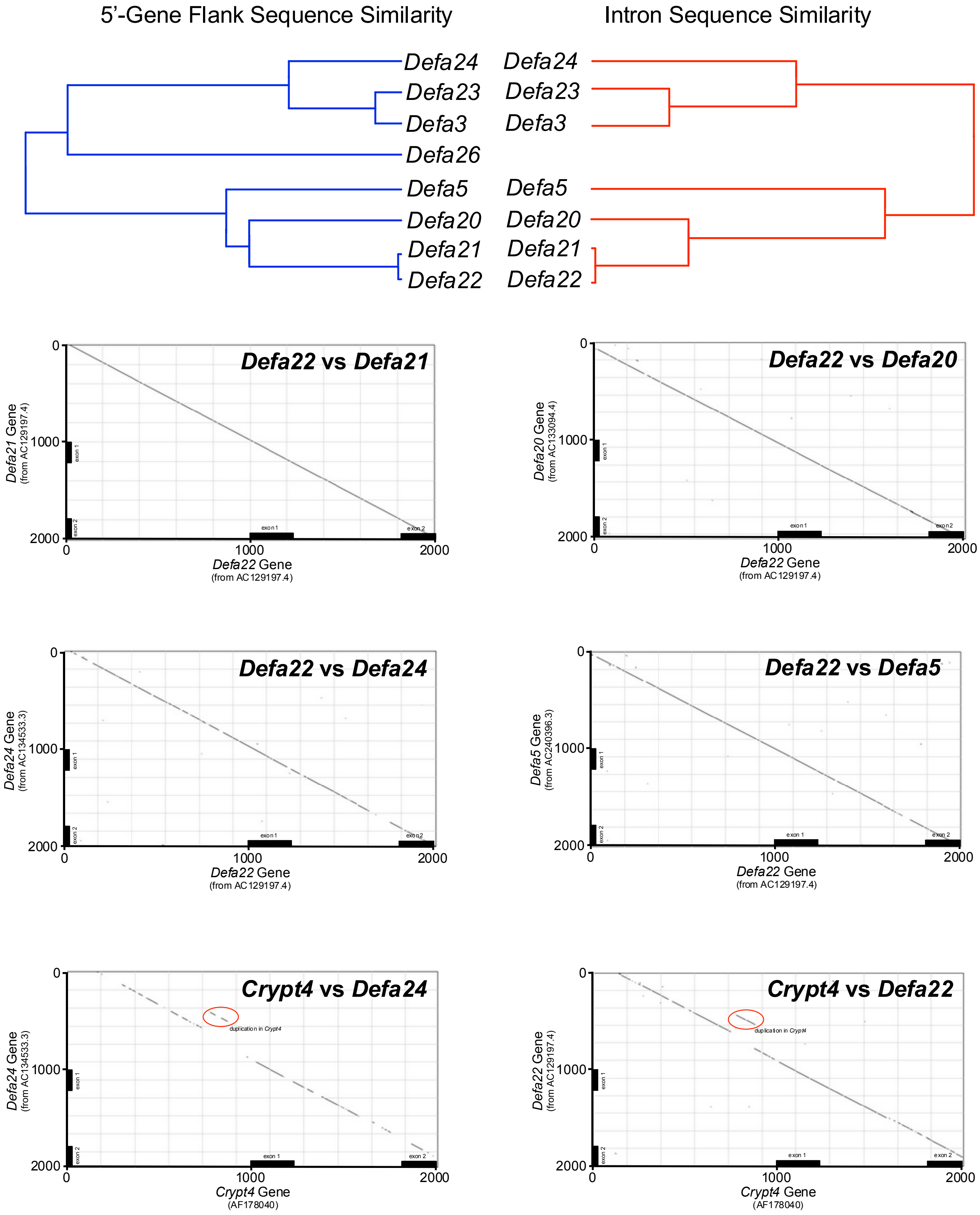

Suppl. Figure 6. *Defa* gene sequence comparisons. The genomic sequences corresponding to mRNA of the index members of the 8 subgroups of C57BL/6 *Defa* paralogs and including ~1 kb of 5'-flanking and 200 BP of 3'-flanking were retrieved from the NCBI database as follows: *Defa3* (AC240396.3, nt 97150 - 99325), *Defa5* (AC240396.3, nt 107100 - 109325), *Defa20* (AC133094.4,

Suppl. Figure 6. *Defa* gene sequence comparisons. (cont'd)
nt 111000 - 108750), *Defa 21* (AC129197.4, nt 61770 - 63775), *Defa 22* (AC129197.4, nt 61780 - 63950), *Defa23* (AC129197.4, nt 91250 - 93450), Defa24 (AC134533.3, nt 135950 - 138125), and *Defa 26* (AC134533.3, nt 19620 - 21630). The accession number for the genomic sequence encoding -defensin cryptdin 4 from 129/SVJ mice is AF78040 [65]. **(A)** Sequence comparison of 5'-flanking sequence (1 kb) of *Defa* genes using ClustalW (MacVector software with default parameters: open gap penalty 15, extended gap penalty 6.7, delay divergent 30%, transitions weighted, UPGMA tree building method, distance uncorrected, best tree). The pattern of similarity was as previously reported [39]. **(B)** Sequence comparison of intron sequence of *Defa* genes using ClustalW (MacVector software with default parameters as in A). The intron of the *Defa26* gene had a 140 BP deletion that placed this gene outside the phylogeny tree of the others and is not included. Note, the pattern of sequence similarity found here for intron sequences, resembles the patterns of sequence similarity both in the 5'-flanking region (A) and in the exons (Fig 1B). **(C-H)** Representative Pustell DNA Matrix pairwise sequence comparisons of genes encoding small intestinal -defensins. *Defa20*, *Defa21*, *Defa22* and *Crypt4* were selected because of their significantly higher expression in the distal small intestine. *Defa 24* was selected because of its high expression uniformly along the length of the small intestine, and *Defa5* was selected because of slightly higher expression distally. Algorithm parameters were: window size 20, minimum percent identity 75%, and hash value 4. All pairwise comparisons of the 8 gene paralogs were performed using 75, 90, and 100% identity parameters exploring possible discriminating matrix patterns for the distal vs the uniform *Defa* genes. The previously identified repeated sequence element in the 5'-flanking region of the *Crypt4* gene [65] was readily detected in this analysis (circle). No similar repeat was detected in the C57BL/6 genes. The data presented are representative of comparisons for all of the *Defa* genes.

Supplementary Table S1

Defa **Paralog Subgroup**	**Members**
Defa3	*Defa3, Defa17*
Defa5	*Defa5*, *Defa34*, *Defa35*, *Defa36, Defa37*
Defa20	*Defa20*, *Defa32*, *Defa33*, *Defa2*
Defa21	*Defa21*
Defa22	*Defa22*
Defa23	*Defa23*, *Defa27*, *Defa31*
Defa24	*Defa24, Defa30*
Defa26	*Defa26,* Gm15292

Supplementary Table S1. Eight *Defa* paralog subgroups of C57BL/6 mice. The subgrouping proposed here was an expansion of the seven groups proposed by Menendez, et al [39] . We were able to establish discriminating assays for *Defa21* and *Defa22*, yielding eight rather than the seven previously described subgroups [39].

Supplementary Table S2

	Defa23	Defa3	Defa26	Defa5	Defa20	Defa22	Defa21
Defa24	86%	86	77	50	36	38	38
Defa23		94	86	83	36	44	41
Defa3			91	53	40	38	41
Defa26				53	40	46	44
Defa5					42	44	44
Defa20						56	54
Defa22							97

Supplementary Table S2. Percent sequence identity of small intestinal α-defensin mature peptides from C57BL/6 mice. Sequences of mature α-defensin peptides are from published studies of others [38,40] and are provided in Supplementary Fig. S3. Percentage of amino acid identity in pairwise comparisons are recorded. Gaps and extensions are recorded as non-identity. No shade represents < 50% identity, light shading 50 - 75% identity, and dark shading > 75% identity.

Supplementary Table S3.

**Gene Targets**	**Primer sequence**	**PCR annealing °C**
Defa3,21,22,23	F:TCCTGCTCACCAATCCTCCAGGT	58°
Defa5,20	F: TCCTGCTCAACAATTCTCCAG	58°
Defa3,23	R: CATATTGCGAACAATTTATTG	58°
Defa5,20,21,22	R: CATATTGCAAACAATTTATTG	58°
Defa24	F: TGCTACTCACCAATCCTCCAGGT<br>R: CATATTGCAAGCAATTTATTG	58°
Defa26	F: TCCTGCTCCCCAATCCCCCAGGT<br>R: CATATTGCGGACAATTTATTG	58°

Supplementary Table S3. Oligonucleotide primer sequences used for PCR amplification and cloning of α-defensin (*Defa*) cDNA standards from C57BL/6 mouse small intestine. The PCR products generated from a small intestinal cDNA template for each of the five reactions (3 & 23, 21 & 22, 5 & 20, 24, 26) were ligated into plasmid DNA and cloned. Sequence analysis of individual plasmid clones identified each of the eight α-defensin cDNAs as follows: *Defa3* = 410 bp; *Defa5* = 409 bp; *Defa20* = 416 bp; *Defa21* = 409 bp; *Defa22* = 409 bp; *Defa23* = 410 bp; *Defa24* = 411 bp; *and Defa26* = 384 bp. Each cloned plasmid DNA was then used as a template to generate quantitative standard curves in subsequent RT-qPCR analysis.

Supplemental References

38 Shanahan, M. T., Tanabe, H. & Ouellette, A. J. Strain-Specific Polymorphisms in Paneth Cell {alpha}-Defensins of C57BL/6 Mice and Evidence of Vestigial Myeloid {alpha}-Defensin Pseudogenes. *Infect Immun* **79**, 459-473 (2011).

39 Menendez, A. *et al.* Bacterial stimulation of the TLR-MyD88 pathway modulates the homeostatic expression of ileal Paneth cell alpha-defensins. *J Innate Immun* **5**, 39-49 (2013).

40 Gulati, A. S. *et al.* Mouse background strain profoundly influences Paneth cell function and intestinal microbial composition. *PLoS ONE* **7**, e32403 (2012).

65 Ouellette, A. J. Peptide localization and gene structure of cryptdin 4, a differentially expressed mouse paneth cell alpha-defensin. *Infect Immun* **67**, 6643-6651 (1999).
